# Downregulation of *PARP1* transcription by CDK4/6 inhibitors sensitizes human lung cancer cells to anticancer drug-induced death by impairing OGG1-dependent base excision repair

**DOI:** 10.1016/j.redox.2017.12.017

**Published:** 2017-12-29

**Authors:** Dominika Tempka, Paulina Tokarz, Kinga Chmielewska, Magdalena Kluska, Julita Pietrzak, Żaneta Rygielska, László Virág, Agnieszka Robaszkiewicz

**Affiliations:** a Department of General Biophysics, Institute of Biophysics, Faculty of Biology and Environmental Protection, University of Lodz, Pomorska 141/143, 90-236 Lodz, Poland; b Department of Molecular Genetics, Institute of Biochemistry, University of Lodz, Pomorska 141/143, 90-236 Lodz, Poland; c Department of Medical Chemistry, Faculty of Medicine, University of Debrecen, Debrecen, Hungary; d MTA-DE Cell Biology and Signaling Research Group, Debrecen, Hungary

**Keywords:** **CDK4/6**, cyclin-dependent kinases 4 and 6, **CDK2**, cyclin-dependent kinase 2, **iCDK4/6**, inhibitor of CDK4/6, **LEE011**, inhibitor of CDK4/6, ribociclib, Kisqali, **PD0332991**, inhibitor of CDK4/6, palbociclib, Imbrance, **NU6140**, cyclin-dependent kinase 2 (CDK2) inhibitor, **PARP1**, poly(ADP-ribose) polymerase 1, **OGG1**, 8-oxoguanine DNA glycosylase, **SMARCA4**, SWI/SNF related, matrix associated, actin dependent regulator of chromatin, subfamily a, member 4, **BER**, base excision repair, **HR**, homologous recombination, **XRCC1**, X-ray repair cross complementing 1, **DNAP β**, DNA polymerase, beta, **PCNA**, proliferating cell nuclear antigen, **MRE11**, double strand break repair nuclease, **RAD51**, RAD51 recombinase, **DNA-PKcs/Ku**, protein kinase, DNA-activated, catalytic polypeptide, **BRCA1**, breast cancer type 1 susceptibility protein, **BRCA2**, breast cancer type 2 susceptibility protein, **Oxo-8-G**, 8-oxo-7,8-dihydroguanosine, **8-oxo-Gua**, 7,8-dihydro-8-oxoguanine, **FapyGua**, 2,6-diamino-4-hydroxy-5-formamidopyrimidine, **iHDAC**, inhibitor of histone deacetylases, **SSBR**, Single-strand break repair, **HDAC1**, histone deacetylase 1, **EZH2**, enhancer of zeste 2 polycomb repressive complex 2 subunit, **iPRC2**, inhibitor of EZH2, UNC1999, **NAC**, N-acetyl-cysteine, **iPARP1**, inhibitor of PARP1, olaparib, **iOGG1**, inhibitor of OGG1, O8, **pCMV3-EMPTY**, A549 cells transfected with control vector, expressing basal level of PARP1, **pCMV3-PARP1**, A549 cells transfected with vector carrying cDNA for PARP1, expressing higher level of PARP1 than pCMV3-EMPTY upon cell cycle arrest in G1, **AP**, apurinic/apyrimidinic site, Cyclin-dependent kinase 4 and 6 (iCDK4/6), Poly(ADP-ribose) polymerase 1 (PARP1), 8-oxoguanine glycosylase (OGG1), Reactive oxygen species (ROS)

## Abstract

Hallmarks of cancer cells include uncontrolled growth and rapid proliferation; thus, cyclin-dependent kinases are a therapeutic target for cancer treatment. Treating non-small lung cancer cells with sublethal concentrations of the CDK4/6 inhibitors, ribociclib (LEE011) and palbociclib (PD0332991), which are approved by the FDA for anticancer therapies, caused cell cycle arrest in the G1 phase and suppression of poly(ADP-ribose) polymerase 1 (*PARP1*) transcription by inducing recruitment of the RB1-E2F1-HDAC1-EZH2 repressive complex to the *PARP1* promoter. Downregulation of PARP1 made cancer cells vulnerable to death triggered by the anticancer drugs (WP631 and etoposide) and H_2_O_2_. All agents brought about redox imbalance and DNA strand breaks. The lack of PARP1 and poly(ADP-ribosyl)ation impaired the 8-oxoguanine glycosylase (OGG1)-dependent base excision DNA repair pathway, which is critical for maintaining the viability of cells treated with CDK4/6 inhibitors during oxidative stress. Upon G1 arrest of PARP1 overexpressing cells, OGG1 formed an immunoprecipitable complex with PARP1. Similar to cells with downregulated PARP1 expression, inhibition of PARP1 or OGG1 in PARP1 overexpressing cells resulted in DNA damage and decreased viability. Thus, PARP1 and OGG1 act in the same regulatory pathway, and PARP1 activity is required for OGG1-mediated repair of oxidative DNA damage in G1-arrested cells. In conclusion, the action of CDK4/6 inhibitors is not limited to the inhibition of cell growth. CDK4/6 inhibitors also lead to accumulation of DNA damage by repressing PARP1 in oxidatively stressed cells. Thus, CDK4/6 inhibitors sensitize G1-arrested cells to anticancer drugs, since these cells require PARP1-OGG1 functional interaction for cell survival.

## Highlights

• CDK4/6 inhibitors arrest cell proliferation in G1 phase.
• iCDK4/6 sensitize cells to DNA damage-induced cell death by repressing PARP1.
• PARP1 is required for OGG1 activity upon growth inhibition.
• OGG1 repairs DNA damaged by WP631, etoposide, or H_2_O_2_ in G1-arrested cells.

## Graphical abstract

**Figure:**
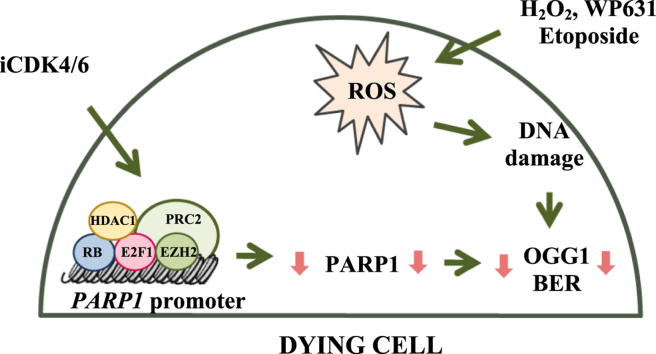

## 1 Introduction

A growing number of people are diagnosed with cancer every year prompting scientists to search for efficient and selective tools for killing transformed and fast proliferating cancer cells. Inhibition of cyclin dependent kinases 4 and 6 (CDK4/6) is a plausible treatment option which has been tested in several clinical trials. Two CDK4/6 inhibitors, LEE011 (ribociclib, Kisqali) and PD0332991 (palbociclib, Imbrance), have recently been approved by the US Food and Drug Administration for the treatment of advanced or metastatic breast cancer in combination with an aromatase inhibitor [1], [2], [3]. Following association with corresponding cyclins, CDKs 4/6 become activated and phosphorylate retinoblastoma protein (RB1), thereby preventing RB1 from binding to and repressing E2F-dependent promoters. E2F-dependent promoters control the transcription of genes encoding *inter alia* proteins capable of promoting cell transition from G1 to S phase. Inhibition of CDK4/6 results in hypophosphorylation of RB1 and assembly of RB1-E2F-based repressor complexes. These complexes consist of histone remodeling enzymes, including histone deacetylases (HDACs), enhancer of zeste 2 polycomb repressive complex 2 subunit (EZH2), and SWI/SNF related, matrix associated, actin dependent regulator of chromatin, subfamily a, member 4 (SMARCA4), which erase transcription activating marks and compact chromatin at gene regulatory elements to inhibit gene expression [4].

Retinoblastoma proteins are involved in the suppression of poly(ADP-ribose) polymerase-1 (PARP1) in human monocytes and in monocytic proliferating precursors upon growth arrest, as we have shown previously [5]. In differentiated cells, the PARP1 promoter was deacetylated by the RBL2-E2F4 dimer-associated HDAC1. On the other hand, in G1-inhibited CD34+ hematopoietic stem cells, RB1-E2F1 recruitment to the promoter was followed by histone deacetylation and trimethylation of H3K27 carried out by HDAC1 (histone deacetylase 1) and EZH2 (enhancer of zeste 2 polycomb repressive complex 2 subunit), respectively. Therefore, hypophosphorylation of retinoblastoma family members by the application of CDK4/6 inhibitors may suppress *PARP1* transcription in fast growing cancer cells. PARP1 protein and protein poly(ADP-ribosyl)ation (PARylation) by PARP1 are involved in the regulation of many intracellular processes, such as signaling, metabolism, gene expression, and DNA repair. Therefore, there is growing interest in the application of PARP1 inhibitors in cancer treatment. PARP1 activation, in response to DNA breaks, leads to mostly auto-PARylation, which acts as a landing platform for the recruitment of DNA repair complexes [6]. These repair pathways include single strand break repair and base excision repair (SSBR and BER: XRCC1, OGG1, DNAP β, DNA ligase III, PCNA, aprataxin, and condensin I), as well as double strand break repair *via* homologous recombination (HR) (active mostly in S and G2 phases) and non-homologous end joining (NHEJ, active in all cell cycle phases) by interacting with MRE11, RAD51, DNA-PKcs/Ku, and DNA ligase IV. PARP1 inhibitors are used in cancer therapies in the setting of BRCA1 (breast cancer type 1 susceptibility protein) or BRCA2 (breast cancer type 2 susceptibility protein) loss (olaparib and rucaparib approved by FDA in patients with HR dysfunction). Clinical trials using PARP1 inhibitors in combination therapy with DNA damaging agents have been conducted recently in HR-deficient and HR-competent tumors [7].

In G1 arrest, repair of double strand breaks shifts from error-free HR to error-prone NHEJ, in which PARP1 plays a suppressive role [8]. PARP1 deficiency releases DNA-PKcs activity, leading to accumulation of DNA errors, and eventually to cell death [9]. Furthermore, by default single strand damages are repaired by BER and SSBR in cells deprived of HR. Quite recently, Noren Hooten described the physical and functional interaction between PARP1 and OGG1 in BER in response to oxidative stress [10]. Oxidative stress, induced by administration of chemical agents, which impair redox homeostasis or by direct cell/tissue irradiation, is often applied as a cancer treatment strategy. OGG1 removes the highly mutagenic 7,8-dihydro-8-oxoguanine (8-oxo-Gua) and 2,6-diamino-4-hydroxy-5-formamidopyrimidine (FapyGua) lesions from DNA, while the loss of OGG1 activity increases the cytotoxicity of multiple therapeutic drugs and IR [11].

Our study indicates that PARP1 is indispensable for OGG1-dependent BER in G1-arrested cells challenged with anticancer drugs, which cause oxidant stress. These drugs, WP631 and etoposide, or the direct oxidant, hydrogen peroxide, caused single (but not double) strand breaks and accumulation of 8-oxo-Gua. PARP1 deficiency, resulting from administration of CDK4/6 inhibitors, impairs OGG1-dependent BER and sensitizes cancer cells to oxidative imbalance-induced death.

## 2 Materials and methods

### 2.1 Materials

The A549 cell line was from ATCC (USA). Cell culture media were purchased from Biowest (CytoGen, Zgierz, Poland). The following antibodies for western blotting were from Santa Cruz Biotechnology (AMX, Łódź, Poland): anti-PARP1 (sc-8007), anti-α-tubulin (sc-5546), anti-pADPribose (10 H, sc- 56198), and gallotannin. TRI Reagent, sodium butyrate (iHDAC), anti-rabbit IgG (A0545) and goat anti-mouse IgG (A4416) (whole molecule)–peroxidase conjugate, hygromycin B, the OGG1 inhibitor O8 were from Sigma Aldrich (Poznan, Poland). The following ChIP grade antibodies were purchased from Cell Signaling Technology (LabJOT, Warsaw, Poland): anti-histone H3 (#4620), anti-H3K27me3 (#9733), anti-RB1 (#9313), anti-E2F1 (#3742), normal rabbit IgG (#2729). Anti-acetyl-histone H3 (Lys9 + Lys14) (PA5-16194), anti-HDAC1 (PA1-860), Texas Red™-X Phalloidin (T7471), Dynabeads*™* Protein G, High-Capacity cDNA Reverse Transcription Kit, SuperSignal™ West Pico Chemiluminescent Substrate, MitoTracker™ Red CMXRos and DAPI were from Thermofisher Scientific (Warsaw, Poland). LEE011 (Ribociclib), PD0332991 (Palbociclib), and NU 6140 were purchased from Cayman Europe (Biokom, Janki/Warsaw, Poland). Kapa Sybr Fast qPCR Master Mix and KAPA HiFi™ HotStart ReadyMix (2×) were purchased from Kapa Biosystems (Polgen, Łódź, Poland). ViaFect™ Transfection Reagent was purchased from Promega (Warsaw, Poland). The human PARP1 Gene cDNA Clone (full-length ORF Clone), expression ready, untagged (HG11040-UT; pCMV3-PARP1) and pCMV3-untagged Negative Control Vector (CV011; pCMV3-EMPTY) were purchased from Hölzel Diagnostika Handels GmbH (Köln, Germany). EvaGreen® Dye, 20× in water was purchased from Biotium (Corporate Place Hayward, USA). Annexin V-FITC Apoptosis Detection Kit I was purchased from Becton Dickinson (Diag-med, Warsaw, Poland). Anti-DNA/RNA Damage antibody [15A3] (FITC) (ab183393) was from Abcam (Bio-Kasztel, Hungary). Human 8-oxoGuanine DNA Glycosylase and 10× REC™ Buffer 6 was from Trevigen (Nordic BioSite OY, Helsinki, Finland).

### 2.2 Cell culture and treatment

A549 cells (non-small lung carcinoma cells derived from primary tumor, passage 10–25) were cultured in DMEM supplemented with 10% FBS and penicillin/streptomycin solution (50 U/ml and 50 µg/ml, respectively). For cell arrest in G1 phase, cells were treated with 2 µM of LEE011 and 1 µM PD0332991 for 48 h. For cell cycle arrest in G2, cells were treated with 10 µM NU6140 for 48 h. To induce redox stress, cells were treated with hydrogen peroxide [H_2_O_2_], WP631, or etoposide. Cell cycle was arrested by treating cells with CDK4/6 inhibitors for 48 h prior to challenging them with oxidative stimuli.

To study the contribution of HDAC(s) and PRC2 to suppression of *PARP1* during growth inhibition, cells cultured in the presence of iCDK4/6 for 24 h were then treated with iHDAC (pan-HDAC inhibitor; 0.5 mM) and iPRC2 (EZH2 inhibitor; 0.125 nM) for an additional 24 h.

### 2.3 Cell viability

Cell viability was determined with MTT assay as previously described [12]. Determination of the mode of cell death was carried out by AnnexinV/propidium iodide staining. Fluorescence was measured by BD LSR II Flow Cytometer according to the protocol described in Robaszkiewicz A et al. [13].

### 2.4 Analysis of the cell cycle

For cell cycle analysis, DNA was stained with propidium iodide followed by flow cytometry measurement of the DNA content as described previously [12].

### 2.5 Evaluation of oxidative stress

Alteration of redox homeostasis was monitored with 2′,7′-dichlorodihydrofluorescein diacetate (H_2_DCF-DA) fluorescent staining as described by Tokarz et al. [14]. Although redox chemistry of hydrolyzed DCFH is complex and includes, among others, oxidation by cytochrome c or redox active metals in the presence of superoxide or O_2_, it mostly indicates oxidant stress [15]. The fluorescence corresponding to probe oxidation was normalized to DNA content, as estimated with propidium iodide. Total thiol content was determined with monobromobiname and normalized to total DNA content as described previously [12].

### 2.6 Generation of PARP1 overexpressing cell lines

A549 cells (passage 4) were transfected with pCMV3-EMPTY or pCMV3-PARP1 expression vector using ViaFect™ Transfection Reagent. In brief, 24 h after plating (approximately 100 000), cells were transfected with the following mixture: 0.1 µg DNA and 0.8 µl ViaFect™ Transfection Reagent in OptiMem, and incubated for 15 min at room temperature prior to cell treatment. Forty-eight hours after transfection, cells were selected with hygromycin B for a month. RNA was extracted with TRI Reagent and additionally treated with DNA-free™ DNA Removal Kit before reverse transcription.

### 2.7 Western blot

Cells were lysed in RIPA buffer (10 mM Tris-HCl pH = 8.0, 140 mM NaCl, 0.5% SDS) supplemented with a proteinase inhibitor cocktail and 1 mM PMSF. For poly-ADP-ribose detection, lysis buffer was also supplemented with PARG inhibitor (1 mM gallotannin). For protein detection, membranes were blocked with 1% BSA in PRB-Tween 20 (0.1%). For poly-ADP-ribose, membranes were blocked with 0.5% gelatin in PRB-Tween 20 (0.1%). Nitrocellulose membranes were stained with primary antibody at 4 °C overnight, then incubated with peroxidase-conjugated anti-rabbit or anti-mouse secondary antibodies (Sigma Aldrich) for at least 2 h at room temperature. Signal was developed using SuperSignal™ West Pico Chemiluminescent Substrate and acquired with ChemiDoc-IT2 (UVP, Meranco, Poznan, Poland).

### 2.8 Gene expression

RNA was extracted with TRI Reagent, reversed transcribed (High Capacity cDNA Reverse Transcription Kit, Thermofisher Scientific), and cDNA was quantified by real-time PCR using Kapa Sybr Fast qPCR Master Mix and CFX96 C1000 Touch real time qPCR detection system (Bio-Rad, Warsaw, Poland). The following primers were used for *PARP1*: Fwd 5′-AAGCCCTAAAGGCTCAGAACG-3′ and Rev 5′-ACCATGCCATCAGCTACTCGGT-3′. PARP1 expression was normalized to *ACTB* (Fwd: 5′-TGGCACCCAGCACAATGAA-3′ and Rev 5′-CTAAGTCATAGTCCGCCTAGAAGCA-3′).

### 2.9 Chromatin immunoprecipitation

Chromatin immunoprecipitation of E2F1, RB1, HDAC1, EZH2, H3, acH3K9/14, and H3K27me3 was carried out according to Wiśnik et al. [5]. The immunoprecipitated DNA (*PARP1* promoter) was quantified with real-time PCR using KAPA HiFi™ HotStart ReadyMix supplemented with EvaGreen® Dye and 7% DMSO. The following primers were used: Fwd 5′-AACGCCCACGGAACCC-3′ and Rev 5′-CTACTAGCTCAGCCCAAGCC-3′.

### 2.10 Protein co-immunoprecipitation

Cells were lysed in IP buffer (50 mM Tris-HCl pH = 7.5, 125 mM KCl, 2.5 mM MgCl_2_, 0.1 mM CaCl_2_, 10% glycerol, 0.1% NP-40). Subsequently, 5 µg rabbit anti-PARP1 antibody was added to 1 mg of nuclear extracts for 3 h (4 °C). 1 h prior to the end of immunoprecipitation, 10 µl of Dynabeads*™* Protein G was added to cell lysates. Beads were washed five times with IP buffer, suspended in 50 µl of RIPA buffer, and mixed with 6× SDS loading buffer. After heating at 75 °C for 10 min, beads were removed on magnetic stand and immunoprecipitated proteins were separated on 10% SDS-gel. Total cell lysate (10%) was loaded on a gel as an input.

### 2.11 Comet assay

Alkaline and neutral version of the comet assay was performed as described by Tokarz et al. [14]. For quantification of 8-oxoguanosine in genomic DNA, agarose-embedded and lysed cells were incubated with human OGG1 in digestion buffer (1 U, 37 °C, 30 min), and then the DNA was subjected to alkaline electrophoresis. The difference in the tail DNA between OGG1 and buffer-treated samples corresponded to the number of OGG1-sensitive sites [16].

### 2.12 Confocal microscopy

After fixation with ice cold methanol for 5 min, cells were permeabilized with 0.1% Triton X100 for 30 min, blocked with 1% BSA/0.1% Triton X100 for 1 h, and stained with a mixture of Texas Red™-X Phalloidin (1:200) and anti-DNA/RNA Damage antibody (1:200) in the blocking solution for 2 h. Nuclei were stained with 2 μg/ml DAPI in PBS. Coverslips were mounted in Mowiol and then viewed with a Leica TCS SP8 confocal microscope. Images were processed with a LAS AF v3.1.3 software.

### 2.13 Statistical analysis

Data is presented as means ± standard errors of the mean (SEM). Student *t*-test was used to compare differences between two means. All bars represent data from at least three independent biological replicates (carried out on different days and with cells of different passage numbers) with two technical replicates each.

## 3 Results

**Fig. 1:**
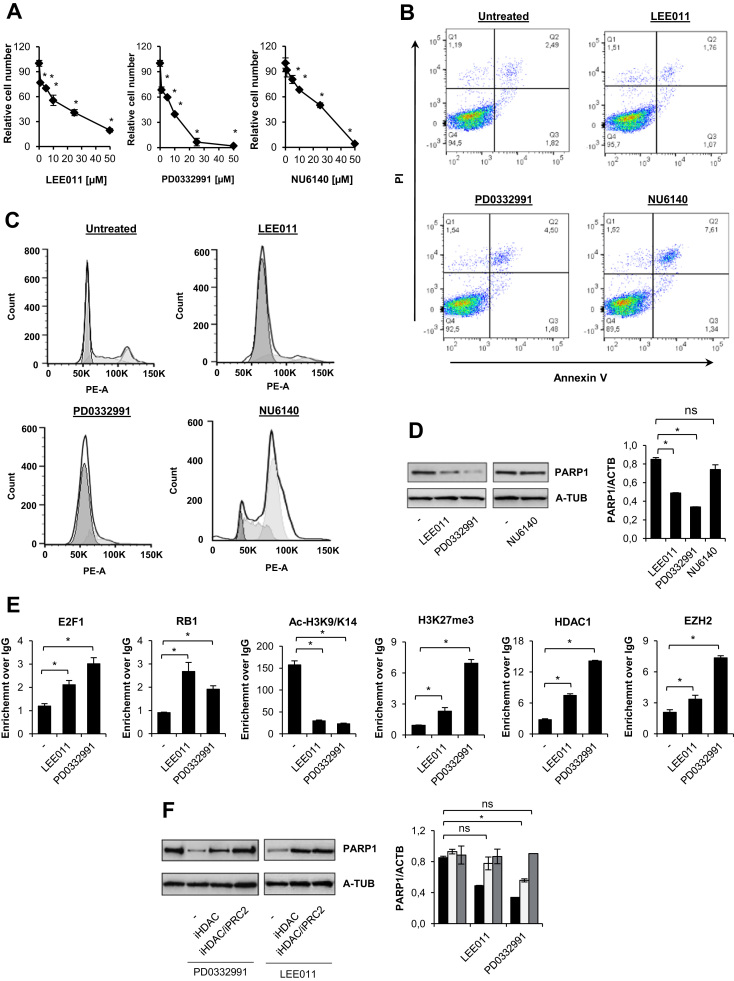
Inhibition of CDK4/6 suppresses *PARP1* expression by inducing formation of E2F1-RB1-HDAC1-PRC2/EZH2 repressive complex. The quantification of living cells by MTT after treatment with CDK4/6 inhibitors LEE011 and PD0332991, as well as with iCDK2 (NU6140) for 48 h is shown (A). The direct toxicity of LEE011 (2 µM), PD0332991 (1 µM), and NU6140 (10 µM) was analyzed by flow cytometry 48 h after incubation with iCDKs (B). Q1 represents necrotic cells, Q2 – late apoptotic, Q3 – early apoptotic, and Q4 – living cells. Cell cycle distribution of cells was determined by flow cytometry after DNA staining with propidium iodide (C). The level of PARP1 protein and mRNA was assessed by western blot and real-time PCR (D), respectively. The composition of repressive complex and histone modifications at the PARP1 promoter were determined by ChIP-qPCR (E). The contribution of HDAC1 and EZH2 to *PARP1* repression was confirmed by treating cells with iHDAC (sodium butyrate − 0.5 mM) and iEZH2 (UNC1999, 125 nM) in addition to LEE011 and PD0332991 (F). PARP1 protein and mRNA recovery was monitored by western blot and real-time PCR, respectively. Bars in the figures represent mean ± SEM, N = 3 (with three technical replicates per experiment for panel A and two technical replicates for other panels), where * indicates p < 0.05. Panels D and F show representative pictures.

To check the direct cytotoxicity of CDK4/6 (LEE011 and PD0332991) and CDK2 (NU6140) inhibitors, we first determined the relative number of living cells after a 48 h treatment with different concentrations (0–50 µM) of the inhibitors. All compounds caused a concentration-dependent inhibition of cell growth with IC70 values of ~ 2 µM, 1 µM, and 10 µM for LEE011, PD0332991, and NU1640, respectively (Fig. 1A). At concentrations corresponding to their IC70 value, CDK inhibitors did not induce cell death (Fig. 1B), but efficiently inhibited cell cycle progression by arresting cells in G1 (LEE011, PD0332991) or in G2 (NU6140) (Fig. 1C). G1, but not G2, arrest substantially downregulated PARP1 expression, both at the protein and at the mRNA level (Fig. 1D). Importantly, of the two CDK4/6 inhibitors, PD0332991 had a stronger suppressive effect on PARP1 expression. An analysis of the *PARP1* promoter revealed that CDK4/6 inhibitors triggered the recruitment of the E2F1-RB1 repressive dimer to the PARP1 promoter (Fig. 1E). Furthermore, E2F1-RB1 binding was followed by loss of the transcription promoting histone mark (acetylation of lysine 9 and 14 at histone 3) and by accumulation of the transcription silencing mark (trimethylation of lysine 27 at histone 3). As expected from the profile of histone marks, *PARP1* promoter was occupied by histone deacetylase 1 (HDAC1, Fig. 1E) and the histone methyltransferase, EZH2, which is a functional enzymatic component of the Polycomb Repressive Complex 2 (PRC2). Notably, PARP1 expression positively correlated with the ac-H3K9/K14 mark and negatively correlated with H3K27me3, HDAC1, and EZH2 recruitment to the *PARP1* promoter. To confirm the functional role of HDAC1 and PRC2 in the regulation of *PARP1* expression, we tested the effects of inhibitors of these enzymes (iHDAC1, 0.5 mM; iPRC2, 0.125 nM). Although the *PARP1* promoter was enriched in EZH2 upon LEE011 administration, inhibition of HDAC1 activity maintained *PARP1* transcription. A further increase in mRNA and protein levels of PARP1 was not observed if PRC2 was also inhibited (Fig. 1F). However, in cells arrested with PD033291, simultaneous inhibition of HDAC1 and PRC2 was required to rescue PARP1 level. Of note, PD0332991 treated cells were characterized by more intense H3K27 trimethylation and higher HDAC1 and EZH2 occurrence when compared to cells treated with LEE011.

### 3.1 Oxidant stress caused by H_2_O_2_, WP631, and etoposide leads to damage of nucleic acids and cell death

**Fig. 2:**
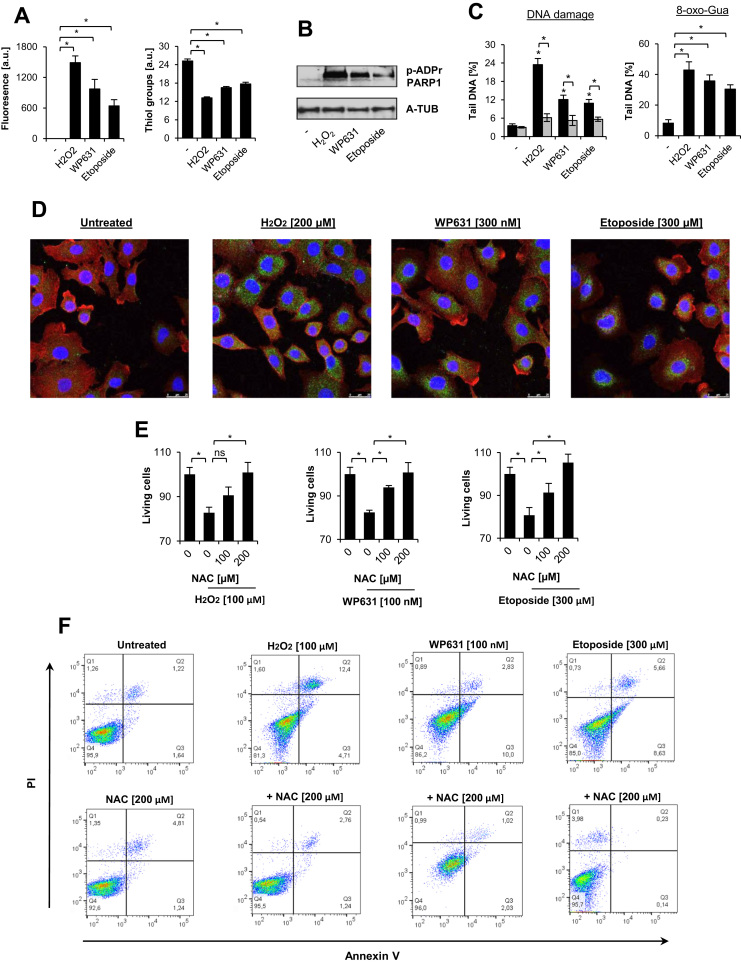
WP631 and etoposide trigger DNA damage and cell death by affecting redox homeostasis. The level of oxidizing molecules and thiols were measured using DCF-DA (5 µM) and monobromobimane (1 µM), respectively, after incubation of cells with H_2_O_2_ (100 µM), WP630 (100 nM), and etoposide (100 µM) for 1 h (A). For the evaluation of protein poly-ADP-ribosylation, cells were incubated as described above and the post-translational modification was monitored by western blot (B). Total DNA damage and accumulation of 8-oxo-guanosine (8-oxo-Gua) was quantified by alkaline comet assay with or without OGG1 treatment (C). Oxidation of RNA (D) was assessed by double staining with anti-RNA/DNA damage (FITC) antibody and with Texas Red®-X phalloidin. The toxicity of H_2_O_2_, WP631, and etoposide and the protective role of N-acetylcysteine (NAC) were measured with MTT testing (E) and by annexin V/PI staining (F). Bars in the figures represent mean ± SEM, N = 3 (with two technical replicates per experiments), where * indicates p < 0.05. Panel B shows representative images.

To evaluate the potential of WP631 and etoposide to trigger redox imbalance, we first measured the production of oxidants and the content of low molecular weight thiols inside cells. We used H_2_O_2_, as a positive control. As shown in Fig. 2A, all of these agents led to oxidation of the fluorescent probe with the following efficacy: H_2_O_2_ > WP631 > etoposide. The thiol content negatively correlated with H_2_DCF-DA fluorescence. A similar profile was observed for PARP1 activation and its auto-PARylation, where H_2_O_2_ stimulated the strongest protein PARylation (Fig. 2B). The PARylation of PARP1 was prevented when cells were pretreated with N-acetyl-cysteine (NAC, Supplement Fig. 1A-C), indicating that oxidative is a bona fide activator of PARylation. A close correlation was also observed between DNA damage and the oxidative stimulus. As for PARylation, all three agents caused DNA damage (accumulation of 8-oxo-Gua and DNA breaks), which was rescued by NAC (Fig. 2C). RNA oxidation was evidenced by detection of Oxo-8-G (8-oxo-7,8-dihydroguanosine) in cytoplasmic RNA (Fig. 2D, extended version in Supplement Fig. 2). Notably, despite the observed impair in redox homeostasis, DNA double-strand breaks remained at very low levels (around 1%) in all samples (Supplement Fig. 1D; a neutral version of the comet assay). Furthermore, cell death induced by all compounds (Fig. 2E-F) was mediated by oxidative signaling, as indicated by the cytoprotective effect of NAC. The treatment of proliferating cells with H_2_O_2_, WP631, and etoposide triggered mostly apoptosis, as indicated by annexin V positivity (Fig. 2F).

### 3.2 PARP1 repression by CDK4/6 inhibitors sensitizes G1-arrested cells to redox imbalance-induced death

**Fig. 3:**
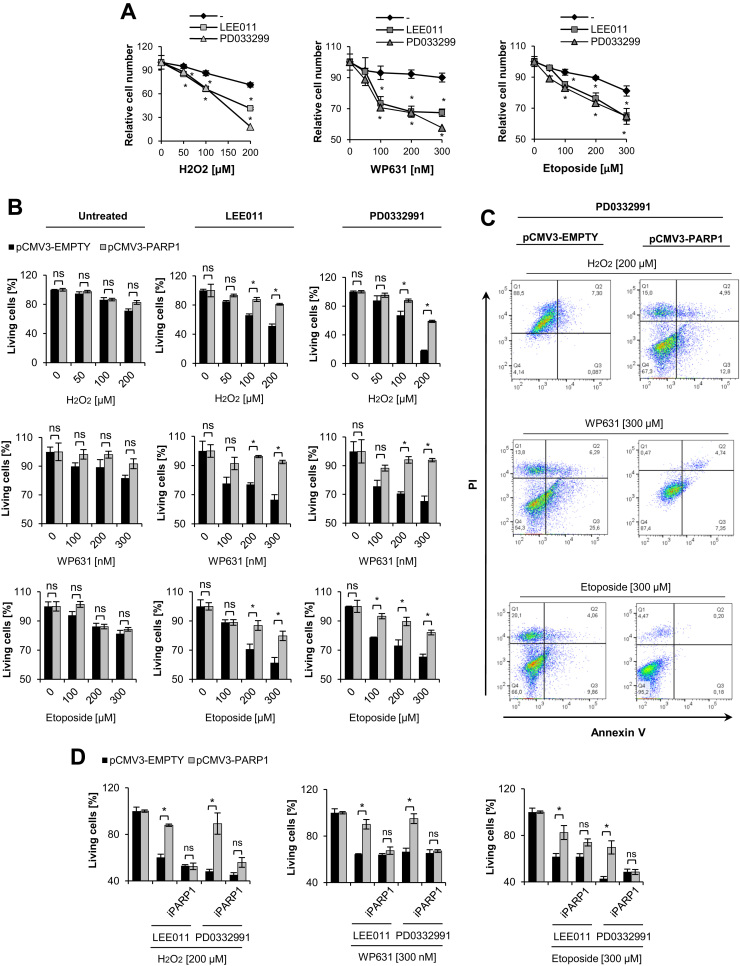
Forced expression of PARP1 rescues G1-arrested cells from death induced by H_2_O_2_, WP631, and etoposide. Sensitivity to death triggered by H_2_O_2_ (0–200 μM), WP631 (0–300 nM), and etoposide (0–300 μM) (24 h post-iCDK4/6 treatment) was measured with MTT testing in proliferating and G1-arrested (LEE011 − 2 µM, PD0332991 − 1 µM) cells (A). The susceptibility of proliferating and G1-arrested pCMV3-EMPTY and pCMV3-PARP1 cells to H_2_O_2_, WP631, and etoposide (24 h exposure) was analyzed with MTT (B) and annexin V/PI staining (C). The effect of the PARP1 inhibitor (iPARP1; olaparib 1 µM) on viability of proliferating and growth arrested pCMV3-EMPTY and pCMV3-PARP1 cells in response to H_2_O_2_, WP631, and etoposide (24 h exposure) was determined with MTT assay (D). Bars in the figures represent mean ± standard error of the mean (SEM), where * indicates p < 0.05; N = 4 independent experiments with three technical replicates per experiment for Fig. A; N = 3 independent experiments in two technical replicates for all other Figures.

As shown in Fig. 3A, arresting cells in the G1 phase with LEE011 and PD0332991 considerably reduced the number of viable cells after treatment with H_2_O_2_ and anticancer drugs compared to proliferating cells. To check if the observed increase in toxicity of oxidative stimuli was caused by PARP1 downregulation, we generated a cell line (pCMV3-PARP1) to keep PARP1 protein and mRNA levels stable upon G1 arrest. The control cell line (pCMV3-EMPTY) displayed PARP1 downregulation after growth inhibition with LEE011 and PD0332991 (Supplement Fig. 3A-B). In contrast to pCMV3-EMPTY, G1-arrested cells overexpressing PARP1 retained PARylation capacity following oxidative stress (Supplement Fig. 3C). Proliferating pCMV3-PARP1 and pCMV3-EMPTY cells showed similar PARP1 levels and sensitivity to the three agents (Suppl. Fig. 3A-B and B). However, upon G1 arrest induced by both LEE011 and PD0332991, pCMV3-PARP1 cells showed significant resistance to the toxic effects of H_2_O_2_ and anticancer drugs (Fig. 3B). Data from MTT tests were confirmed by annexin V/PI staining of cells (Fig. 3C). Notably, in contrast to proliferating cells, pCMV3-EMPTY cells treated with PD0332991 and DNA damaging agents showed a substantial increase in single PI-positive cells (Fig. 2F, 3C). These data suggest that cell growth inhibition followed by *PARP1* repression made cells vulnerable to necrosis, while sustained PARP1 expression protected cells in G1-arrest from DNA damage-induced cell death.

To verify the contribution of PARylation to the protection of growth-inhibited cells from DNA damage-induced cytotoxicity, we tested the effect of the PARylation inhibitor, olaparib (iPARP1) in pCMV3-PARP1 cells. Olaparib alone or in combination with CDK4/6 inhibitors did not affect cell viability (Supplement Fig. 3D). However, inhibition of PARP1 activity completely abolished the protective effect of PARP1 overexpression on cell survival in LEE011/PD0332991 and cytotoxin-treated cells (Fig. 3D).

### 3.3 PARP1 is required for OGG1-dependent BER in G1-arrested cells and protects cells from toxicity caused by anticancer drugs

**Fig. 4:**
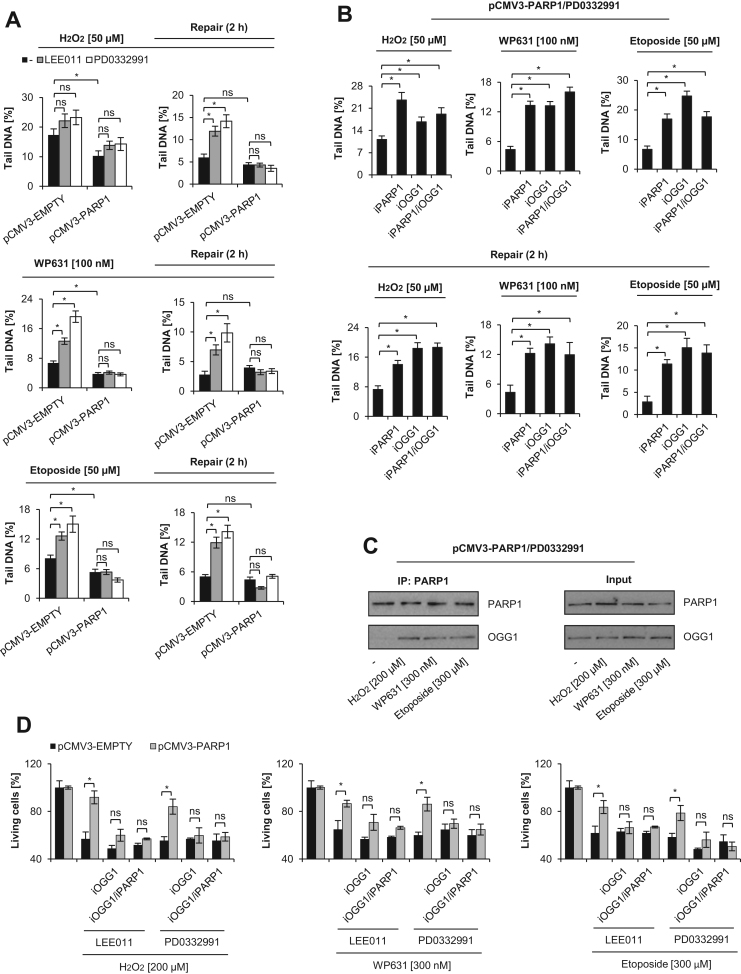
PARP1 repression impairs OGG1-mediated repair of DNA lesions. Alkaline comet assay was used to quantify the level of DNA strand breaks in proliferating and G1 arrested cells treated with the cytotoxic agents (H_2_O_2_ − 50 μM; WP631 – 100 nM; and etoposide − 75 μM) (A). In the left panels, cells were treated with H_2_O_2_ for 15 min and with WP631 or etoposide for 2 h. Afterwards, toxic agents were removed from the culture by washing with growth medium and cells were incubated at 37 °C for another 2 h (repair; right panels). To test the contribution of PARP1 and OGG1 activity to the repair of oxidative DNA damage in G1-arrested pCMV3-PARP1 expressing cells (B, upper panels), cells were preincubated with iPARP1 (olaparib, 1 µM) or iOGG1 (O8, 10 µM) for 1 h. Afterwards, cells were treated and analyzed as described in A. In growth-arrested pCMV3-PARP1 cells treated with the toxic agents, PARP1-OGG1 physical interaction was detected by co-immunoprecipitation (C). The effect of PARP1 and OGG1 inhibition on the viability of G1-arrested cells was determined with MTT testing (D). iPARP1 and iOGG1 were added to cells 1 h prior to addition of H_2_O_2_, WP631, and etoposide. Bars in the figures represent mean ± SEM, N = 3 (with two technical replicates per experiment), where * indicates p<0.05;. Panel C shows representative images.

Since H_2_O_2_, WP631, and etoposide caused DNA damage, and PARP1 was reported to protect the genome, we set out to determine the effects of *PARP1* repression on DNA damage repair mechanisms in growth-arrested cells. As shown in Fig. 4A, even a short incubation with H_2_O_2_ led to statistically significant differences in DNA strand break levels between pCMV3-EMPTY and pCMV3-PARP1 cells. The discrepancy became even more distinct after an additional 2 h incubation in the absence of H_2_O_2_. PARP1 overexpressing cells challenged with H_2_O_2_ completely repaired DNA strand breaks regardless of G1 inhibition. On the contrary, pCMV3-EMPTY cells were defective in DNA repair and the accumulation of DNA damage correlated with the repression of *PARP1* in response to LEE011 and PD0332991. A similar profile of interdependence between PARP1 level and the extent of DNA strand breaks was observed in cells treated with WP631 and etoposide even before the withdrawal of the toxic agent (Fig. 4A, left panels). Although some decrease in DNA damage was found after WP631 removal, the pCMV3-EMPTY cells treated with LEE011 and PD0332991 displayed a high number of genomic lesions. Etoposide removal did not affect the level of DNA breaks in these cells (Fig. 4A, right panels). Neither WP631 nor etoposide brought about accumulation of DNA lesions in pCMV3-PARP1 cells.

Since these genotoxic agents cause oxidation of guanine, we checked if OGG1 contributed to DNA break repair and if PARP1 is required for OGG1 activity. For this purpose, we pretreated G1-arrested (PD0332991) pCMV3-PARP1 cells with PARP1 (iPARP1 - olaparib) and OGG1 (iOGG1 – O8) inhibitors (separately and in combination). None of these compounds caused DNA lesions in cells (Suppl. Fig. 3E). However, inhibition of either one or both enzymes led to a substantial and comparable accumulation of DNA strand breaks triggered by the three genotoxic agents (Fig. 4B, upper panels). Similar to pCMV3-EMPTY cells arrested with LEE011 and PD0332991, inhibition of PARP1 or OGG1 maintained DNA lesions in pCMV3-PARP1 cells after withdrawal of the toxic substances (Fig. 4B, lower panels). These data indicate that PARylation is indispensable for the OGG1-dependent repair of these DNA lesions in G1-arrested cells. The lack of synergism between iPARP and iOGG1 suggests that both enzymes act in the same regulatory pathway. The immunoprecipitation of PARP1 in growth inhibited (PD0332991) pCMV3-PARP1 cells revealed a functional interaction between these two enzymes (Fig. 4C). To verify the possible role of OGG1 in cell survival in the presence of PARP1 and their mutual cross-talk, we compared the viability of G1-arrested pCMV3-EMPTY and pCMV3-PARP1 cells incubated with iOGG1 and iOGG1/iPARP1 prior to cell exposure to anticancer drugs and H_2_O_2_ (Fig. 4D). Similar to PARP1 inhibition, OGG1 inhibition sensitized PARP1 overexpressing cells and reduced the number of living cells to the level observed in the corresponding pCMV3-EMPTY cells. Simultaneous inhibition of both enzymes did not lead to further increase in cell death. Of note, neither iPARP1 nor iOGG1 affected the survival of pCMV3-EMPTY cells upon alteration of redox homeostasis. Altogether these data indicate that *PARP1* repression in response to growth inhibition impairs OGG1–dependent BER, thereby leading to increased sensitivity to the cytotoxic effects of anticancer drugs and H_2_O_2_.

## 4 Discussion

The discovery of the role of CDK 4/6 in cell cycle progression and development of inhibitors allowed scientists to investigate the effects of CDK4/6 inhibitors in fast-proliferating cancer cells. Our study shows that CDK4/6 inhibitors, even at sublethal concentrations, cause a considerable decrease in *PARP1* transcription, in addition to suppressing lung cancer cell growth. Although both inhibitors arrested the cell cycle in G1 phase, PD0332991 suppressed PARP1 expression more than LEE011 (Fig. 1D). The G2 phase blocker NU1640 also stopped the cell cycle, but did not affect PARP1 expression. This finding indicates that PARP1 downregulation is a specific event of the G1 phase. These findings are in line with our previous observations made in THP-1 cells; cells arrested in G1 (with mimosine), but not in G2 (with nocodazole), lost their ability to maintain high *PARP1* levels.

A different composition of repressive complexes at the *PARP1* promoter may explain the differences in *PARP1* suppression after treatment with these CDK4/6 inhibitors. Both inhibitors induced the assembly of the same repressive complex (E2F1-RB1-HDAC1-EZH2). However, enrichment of these factors (except RB1) was higher in cells arrested with PD0332991. Since histone acetylation was similar, EZH2 and H2K27me3 are likely the key repressors of *PARP1* expression. This was confirmed with iHDAC and iEZH2, because only PD0332991 treated cells required both inhibitors for PARP1 derepression. Interestingly, the level of E2F1 at the *PARP1* promoter correlated with both HDAC1 and EZH2. E2F1 has also been documented to repress genes independent of RB1, and E2F1 activity is likely to be controlled by multiple factors. Previously, E2F1 was shown to directly repress the gene of the anti-apoptotic protein Mcl-1. The Rb1-independent interaction of E2F1 with the class III histone deacetylase, SirT1 and HDAC1, through KAP1 has also been demonstrated [17], [18]. Furthermore, the binding of TopBP1 to E2F1 was found to recruit SMARCA4 and SMARCA2 (the components of the SWI/SNF chromatin-remodeling complex) and repress some of the E2F1-dependent genes [19], [20]. Thus, overrepresentation of E2F1 at the PARP1 promoter in PD0332991-treated cells may be responsible for the increased occurrence of HDAC1 and EZH2. However, the mechanisms responsible for the strong accumulation of E2F1 on the *PARP1* promoter remain unknown and may display promoter and signal specificity.

Since CDK4/6 inhibitors efficiently repress PARP1 expression, their therapeutic application should be reevaluated and extended from simple growth inhibition to combination therapies involving DNA damaging agents. In this novel scenario, iCDK4/6-induced PARP1 depletion could sensitize growth arrested cancer cells to cytotoxic agents. The hitherto clinical application of PARP inhibitors is based on the PARP-BRCA synthetic lethality paradigm, exploiting homologous recombination repair deficiency of tumors (*e.g.* due to germline BRCA1/2 mutation or a functional BRCA-ness phenotype) [21]. The latest approaches in synthetic lethality aim to use various PARP1 inhibitors (talazoparib, veliparib, rucaparib, niraparib) for the treatment of cancers with defects other than BRCA1 deficiencies, including defects in *PALB2*, *FANCD2*, *RAD51*, *ATM*, *MRE11*, *p53*, *XRCC1* and *LSD1* [22]. The knowledge on how to suppress *PARP1* transcription extends the list of potential partners to be targeted in order to develop synthetic lethality. One must be cautious, however, not to equate PARP1 repression with PARP1 inhibition. When PARP1 is inhibited the enzyme gets trapped at DNA breaks and this feature is considered important to the clinical activity of PARP inhibitors. Nonetheless, it may be worthwhile to test the efficacy of combination therapies with iCDK4/6 and DNA damaging chemotherapeutics or irradiation.

Although CDK4/6 inhibitors sensitized A549 lung cancer cells to death induced by the anticancer drugs, WP631 and etoposide, *in vitro*, the idea of combining *PARP1* repression should be considered on a case by case basis and particular attention should be paid to the type of cancer and death-inducing agents. One should always bear in mind that PARP1 and PARylation regulate many intracellular processes. For example, intense PARP1 activation (*e.g.* in response to severe DNA damage) impairs cellular metabolism by NAD^+^ consumption. Therefore, PARP1 deficiency or inhibition have been documented to prevent energetic failure (NAD^+^ and ATP depletion) [23], [24]. Over-activation of PARP1 may also function as a necrotic cell suicide mechanism in cells exposed to intense oxidant stress. However, stimuli applied in our current study caused less severe, repairable DNA damage [25], [26]. At the genomic level, PARP1 was documented to regulate transcription of both tumor suppressors and oncogenes and effectors of metastatic processes (E-cadherin, fibronectin). PARP1 was also shown to co-operate with nuclear receptors (estrogen, progesterone and androgen receptors) in cancer cells [27]. Therefore, genomic and transcriptome profiles should be checked before the administration of PARP1 suppressive agents such as CDK4/6 inhibitors.

Another limitation in using the combinatory treatment (CDK4/6 inhibitors and DNA damaging agents) is the deficiency of RB1 in some primary cancer cells, which has been linked to poor prognosis and altered response to anticancer treatment [28], [29], [30]. The relatively fast proliferating cancer cell line (A549) used in this study is positive for both PARP1 and RB1 (as confirmed by western blots – PARP1 and chromatin immunoprecipitation in G1 arrested cells – RB1). Thus, it allowed us to create a functional link between RB1, PARP1 and OGG1. The lack of any of these three proteins or likely mediators between RB1 and PARP1 or PARP1 and OGG1 precludes the practical application of the approach presented in this study. Previous studies have documented that *RB1* is mutated in most retinoblastomas, osteosarcomas, and small-cell lung cancers, but also in other cancer types at lower frequencies [31]. RB1 deficiency makes cancer cells resistant to CDK4/6 inhibitors, but may also prevent efficient *PARP1* gene targeting.

An interesting finding in our current work shed light on the relationship of PARP1 and OGG1. Our findings suggest that PARP1 is required for proper functioning of OGG1 in G1-arrested cells. However, Noren Hooten et al. reported a bidirectional interplay between these two proteins, identifying a direct, hydrogen peroxide-enhanced binding of the proteins. Hooten found that OGG1 stimulated PARP1, while PARP1 activity inhibited OGG1 function [10]. That observation was made in proliferating HeLa cells, suggesting that PARP1-OGG1 cross-talk is cell type or cell cycle progression dependent, rather than oxidant dependent, since the interactions occurred in H_2_O_2_ treated cells.

PARP1 downregulation in G1-arrested cells or inhibition of PARP activity in PARP1 overexpressing cells phenocopied the effect of OGG1 inhibition on both DNA damage repair and cell viability. Thus, poly(ADP-ribosyl)ation was indispensable for the maintenance of genome integrity and cell survival. In human lens epithelial cells (HLE-B3) challenged with hydrogen peroxide or the redox cycling quinone menadion, acetylation of K338/K341 lysine residues in OGG1 by EP300 was required for activity of OGG1 [32]. Importantly, our recent findings indicate that PARP1 recruits EP300 to some gene promoters. Thus, it sounds plausible to hypothesize that in our model PARP1 may recruit EP300 to the site of oxidative DNA damage where it activates OGG1 to stimulate DNA repair [33]. However, this hypothesis requires experimental confirmation.

OGG1 functions as a DNA glycosylase and excises 8-oxoguanine from DNA, leaving behind an apurinic/apyrimidinic (AP) site. Thus, the observation that OGG1 inhibition in PARP1 overexpressing and G1-arrested cells led to accumulation of DNA strand breaks was somewhat unexpected. These data suggest that OGG1 either contributes to the removal of single strand lesions or another less specific DNA glycosylase or lyase takes over the function of OGG1. The first option is supported by the *in vitro* findings showing that 3′-end 8-oxoguanine, which can arise as a consequence of ionizing radiation and as a result of misincorporation of 8-oxo-dGMP, required APE1 for the proper ligation of DNA ends [34]. As OGG1 is involved in the 8-oxo-Gua removal *in vivo*, the inhibition of OGG1 may maintain strand breaks carrying 8-oxo-Gua at the lesions’ end(s).

Quite surprisingly, the deficiency of only OGG1 activity in PARP1 overexpressing and G1-arrested cells caused the loss of cell viability after treatment with DNA damaging agents. A similar observation was made in KG-1 human leukemia cells with an OGG1 mutation. In these cells, loss of OGG1 activity resulted in the accumulation of 8-hydroxyguanine in DNA and enhanced sensitivity to irradiation [35]. On the other hand, OGG1 overexpression protected human embryonic kidney cells from platinum toxicity [36]. Furthermore, *Ogg1*^−/−^ mice displayed lower repair capacity for 8-oxo-Gua and increased mutagenesis frequencies due to GC to TA transversions [37]. Since base excision repair of 7,8-dihydro-8-oxo-2′-deoxyguanosine is indispensable for cell survival in certain conditions, inhibition of OGG1 may become a novel anticancer treatment modality either in monotherapy or in combination with DNA damaging agents.

In summary, inhibitors of CDK4/6 sensitize lung cancer cells to oxidative stress-inducing anticancer drugs by creating a functional link between RB1, PARP1, and OGG1. By repressing PARP1, CDK4/6 inhibitors indirectly impair OGG1-dependent base excision repair.
