# Supplementary material for: Downregulation of PARP1 transcription by CDK4/6 inhibitors sensitizes human lung cancer cells to anticancer drug-induced death by impairing OGG1-dependent base excision repair
Source: Redox Biol. 2017 Dec 29;15:316–26. doi: 10.1016/j.redox.2017.12.017 (PMC5975074; doi:10.1016/j.redox.2017.12.017)
Supplement: Supplementary file 1 — Supplementary material [file mmc1.pdf]

Supplementary Fig. 1

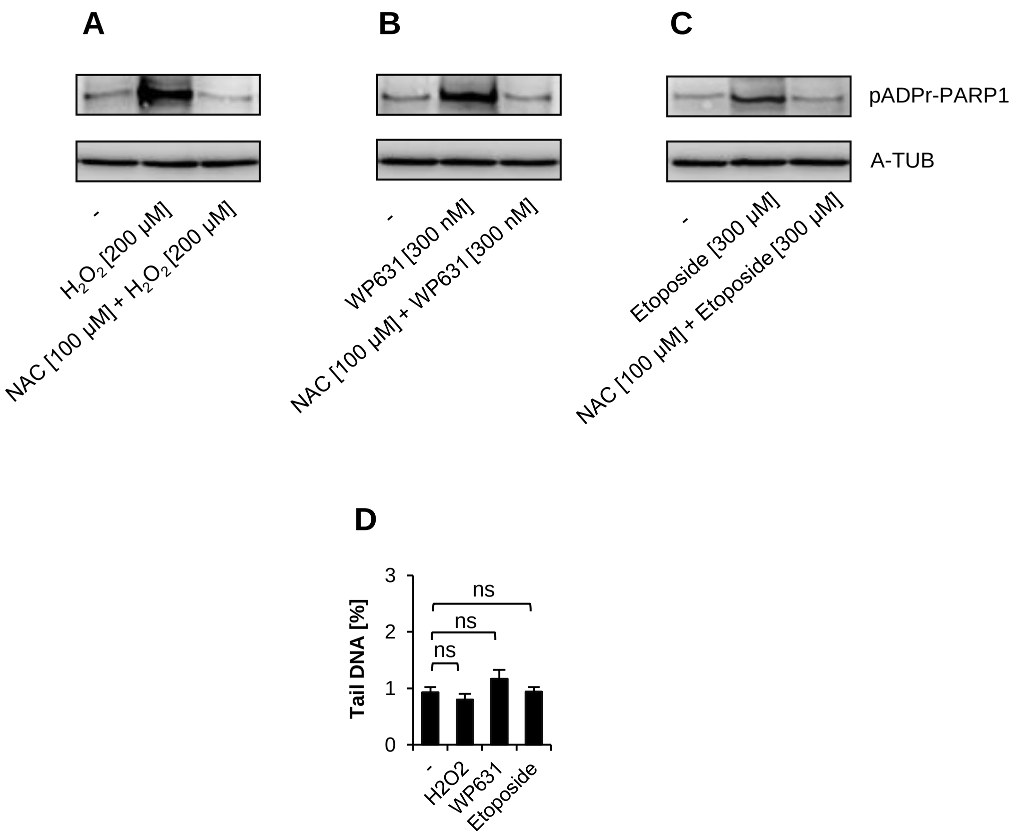

Supplementary Fig. 1: The ROS-induced protein poly(ADP-ribosyl)ation initiated by the cell treatment with H_2O_2 (15 min), WP631 and etoposide (2 h) was inhibited by cell pre-incubation with N-acetylcystein (NAC) for 1 h (A-C). Double strand breaks (D) were quantified by neutral version of the comet assay in cells treated with oxidants as described in A-C.

Supplementary Fig. 2

Untreated | H_2O_2 [200 µM] | WP631 [300 nM] | Etoposide [300 µM]

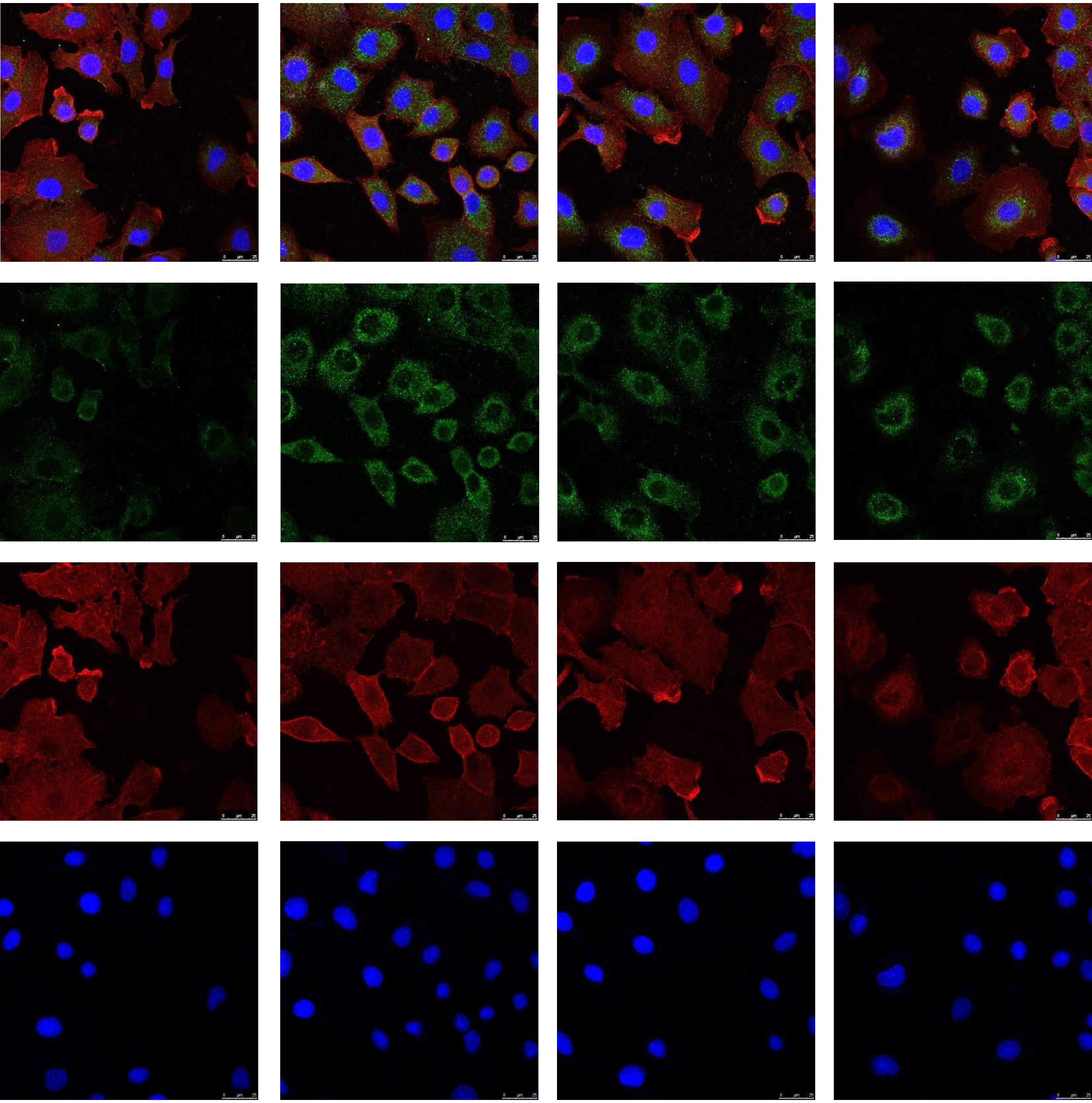

Supplementary Fig. 2: The detection of 8-oxo-7,8-dihydroguanosine (Oxo-8-G) after cell incubation with oxidants for 2 h was carried out by tripple cell staining with anti-DNA/RNA damage antibody (green), Texas Red®-X phalloidin (red) and DAPI (blue).

Supplementary Fig. 3

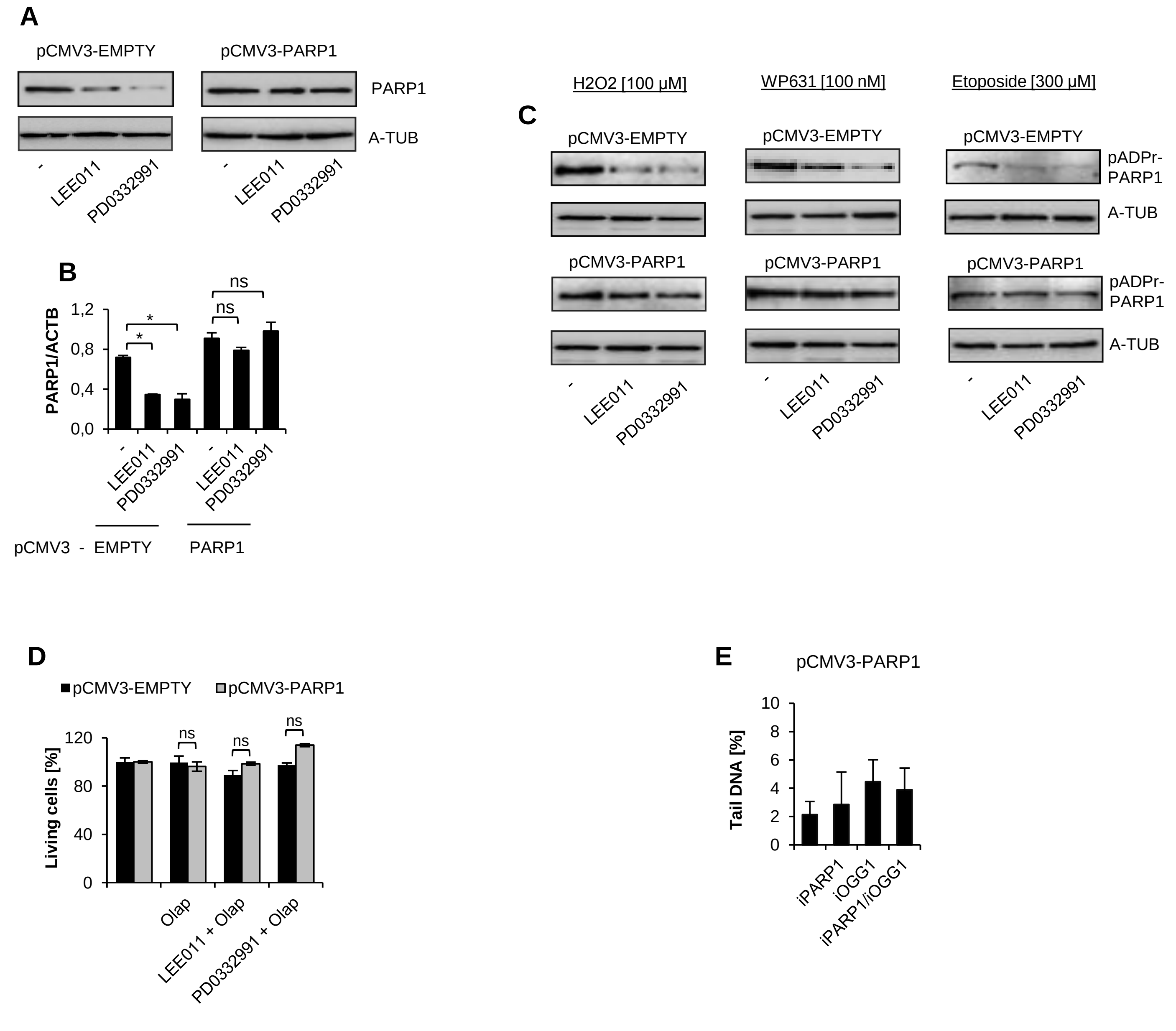

Supplementary Fig. 3

The maintenance of PARP1 expression at protein (A, western blot) and mRNA (B, real-time PCR) upon arresting cell growth was achieved by generating cell lines carrying pCMV3-EMPTY and pCMV3-PARP1. The extent of protein poly-ADP-ribosylation in pCMV3-EMPTY and pCMV3-PARP1 cells treated with CDK4/6 inhibitors and oxidants was monitored by western blot (C). Alpha-tubulin (A-TUB) was used as a control. The effect of PARP1 inhibitor (iPARP1; olaparib – 1 µM) on cell viability was determined with MTT assay (growth-arrested cells were incubated for another 24 h with olaprib). The DNA damage after G1-arrested pCMV3-PARP1 cell treatment with iPARP1 (1 µM) and iOGG1 (10 µM) for 2 h was quantified by alkaline version of comet assay (E).
